# Supplementary material for: Mutational scanning reveals the determinants of protein insertion and association energetics in the plasma membrane
Source: eLife. 2016 Jan 29;5:e12125. doi: 10.7554/eLife.12125 (PMC4786438; doi:10.7554/eLife.12125)
Supplement: Supplementary file 1. — The insertion data (Figure 2b, points) were fitted to the following fourth-order polynomial, where Z is given in Å (Z = 0 at the membrane midplane) and ΔG is in kcal/mol:∆Ginsertionapp=a0Z4+a1Z3+a2Z2+a3Z+c Parameter c is the value of insertion at the membrane midplane. The right-hand column reports the r2 value for the polynomial fit to the data points. The fit for Glu (E) is poor, reflecting the flatness of the Glu profile (Figure 2b). DOI: http://dx.doi.org/10.7554/eLife.12125.016 [file elife-12125-supp1.docx]

**Tables**

| Amino acid | $a_{0}$(x10^-5^) | $a_{1}$(x10^-4^) | $a_{2}$ (x10^-2^) | $a_{3}$(x10^-2^) | $c$ | *r*^2^ |
| --- | --- | --- | --- | --- | --- | --- |
| R | 0.60 | -1.22 | -0.44 | 9.78 | 0.79 | 0.98 |
| K | -0.16 | -1.18 | 0.00 | 9.81 | 1.51 | 0.94 |
| D | -0.29 | -1.82 | 0.22 | 3.67 | 1.54 | 0.81 |
| E | -0.11 | 0.05 | -0.02 | -0.44 | 1.83 | 0.17 |
| H | -0.04 | 2.11 | -0.31 | -2.89 | 1.85 | 0.89 |
| N | 0.37 | -0.20 | -0.24 | -1.25 | 1.28 | 0.75 |
| Q | -0.21 | 1.29 | 0.17 | -2.41 | 0.97 | 0.43 |
| S | -0.15 | 0.89 | 0.12 | -1.64 | 0.09 | 0.62 |
| T | 0.14 | -0.19 | -0.13 | 2.76 | 0.83 | 0.79 |
| P | -0.14 | 0.71 | -0.11 | -1.64 | 1.87 | 0.72 |
| C | -1.16 | -1.06 | 0.80 | 0.09 | -0.12 | 0.94 |
| G | 0.37 | 0.10 | -0.47 | 0.23 | 1.58 | 0.88 |
| A | 0.00 | 0.00 | 0.00 | 0.00 | 0.00 | ------ |
| V | -0.62 | -0.13 | 0.30 | -1.42 | -0.60 | 0.68 |
| I | -0.97 | -0.72 | 0.63 | 2.03 | -1.57 | 0.86 |
| L | -1.78 | 0.74 | 0.87 | -5.08 | -1.92 | 0.89 |
| M | -1.12 | 0.08 | 0.64 | -2.81 | -0.80 | 0.86 |
| F | -1.54 | -1.14 | 1.10 | 3.44 | -1.63 | 0.92 |
| Y | -0.80 | 1.87 | 0.33 | -5.68 | 0.84 | 0.82 |
| W | -0.65 | 1.20 | 0.49 | -4.86 | -0.35 | 0.81 |
