# Supplementary material for: Mutational scanning reveals the determinants of protein insertion and association energetics in the plasma membrane
Source: eLife. 2016 Jan 29;5:e12125. doi: 10.7554/eLife.12125 (PMC4786438; doi:10.7554/eLife.12125)
Supplement: Supplementary file 2. — For each membrane-spanning wild-type segment we optimized the selection stringency. Mutated sequences were used as negative controls in experiments to identify optimal selection regimes, where the difference in bacterial growth between wild-type sequence and mutant was largest. DOI: http://dx.doi.org/10.7554/eLife.12125.017 [file elife-12125-supp2.docx]

|  | CLS | | GpA | | ErbB2 | |
| --- | --- | --- | --- | --- | --- | --- |
| wild-type sequence | PLFIPVAVMVTAFSGLAFIIWLATSGG | | LIIFGVMAGVIGTILI | | LTSIISAVVGILLVVVLGVVFGILI | |
| Mutant sequence | PLFIPVAVM**R**TAFSGLAFIIWLATSGG  (V304R) | | LIIFGVMA**I**VIGTILI  (G83I) | | LTSIISAVV**V**ILLVVVLGVVF**V**ILI  (G636V;G648V) | |
| Antibiotic concentration used in experiments  (μg/ml) | AMP* | AMP/CAP** | AMP | AMP/CAP | AMP | AMP/CAP |
|  | 400 | 400/20(library)  400/30(WT) | 100 | 100/7.5 | 100 | 100/75 |
